# Effect of Strain Rate on Nano-Scale Mechanical Behavior of A-Plane ($11\bar{2}0$) ZnO Single Crystal by Nanoindentation

**DOI:** 10.3390/mi14020404

**Published:** 2023-02-08

**Authors:** Xiaolin Zhu, Jijun Li, Lihua Zhang, Fengchao Lang, Xiaohu Hou, Xueping Zhao, Weiguang Zhang, Chunwang Zhao, Zijian Yang

**Affiliations:** 1 College of Science, Inner Mongolia University of Technology, Hohhot 010051, China; 2 School of Mechanical and Energy Engineering, Shanghai Technical Institute of Electronics & Information, Shanghai 201411, China; 3 College of Science and Technology, Inner Mongolia Open University, Hohhot 010011, China; 4 College of Arts and Sciences, Shanghai Maritime University, Shanghai 201306, China; 5 Test Center, Inner Mongolia University of Technology, Hohhot 010051, China; 6 School of Materials Science and Hydrogen Energy, Foshan University, Foshan 528000, China

**Keywords:** a-plane ($11\bar{2}0$) ZnO single crystal, strain rate, nano-scale mechanical behavior, nanoindentation, load–indentation-depth curve, pop-in, hardness, Young’s modulus

## Abstract

In this study, nanoindentation tests at three different strain rates within 100 nm indentation depth were conducted on an a-plane ($11\bar{2}0$) ZnO single crystal to investigate the effect of strain rate on its nano-scale mechanical behavior. The load–indentation-depth curves, pop-in events, hardness and Young’s moduli of an a-plane ($11\bar{2}0$) ZnO single crystal at different strain rates were investigated at the nano-scale level. The results indicated that, with the indentation depth increasing, the load increased gradually at each maximum indentation depth, *h*_ma_, during the loading process. A distinct pop-in event occurred on each loading curve except that corresponding to the *h*_max_ of 10 nm. The applied load at the same indentation depth increased with the increasing strain rate during the nanoindentation of the a-plane ($11\bar{2}0$) ZnO single crystal. The higher strain rate deferred the pop-in event to a higher load and deeper indentation depth, and made the pop-in extension width larger. The hardness showed reverse indentation size effect (ISE) before the pop-in, and exhibited normal ISE after the pop-in. Both the hardness and the Young’s modulus of the a-plane ($11\bar{2}0$) ZnO single crystal increased with the increasing strain rate, exhibiting the positive strain-rate sensitivity.

## 1. Introduction

The zinc oxide (ZnO) single crystal is regarded as a promising third-generation semiconductor, owing to its wide band gap (~3.37 eV), high exciton binding energy (~60 meV), high thermal conductivity, high chemical stability, piezoelectricity and optical transparency [1,2,3,4,5]. Nowadays, the ZnO single crystal has broad application prospects in piezoelectric transducers, surface-acoustic-wave filters, short-wavelength photoelectric devices, gas sensors, transparent electrodes and solar cells [6,7,8,9,10].

Tremendous efforts have been devoted to studying the growth and electrical/optical properties of the ZnO single crystal [11,12,13,14], while less attention has been paid to its mechanical properties. During the processes of fabrication and service of ZnO-single-crystal devices, external contact stress will be applied to the ZnO single crystal, which could result in surface contact damage, and significantly degrade the performance of these devices [15,16]. Therefore, a comprehensive understanding of the mechanical properties of the ZnO single crystal can be especially important for the performance improvement and reliability assessment of ZnO-single-crystal devices. Meanwhile, with the rapid development of nanotechnology, the semiconductor-device integration is improving continuously, and the characterization of mechanical behaviors of semiconductors on the micro- and nano-scale is becoming more urgent and necessary.

In recent years, nanoindentation technology has become a very powerful tool for studying mechanical response of materials on a micro- and nano-scale level, due to its convenience in sample preparation, small test area, high resolution and non-destructive featurse [17,18,19,20]. The mechanical parameters of a material such as hardness, Young’s modulus and fracture toughness on the micro- and nano-scale can be obtained from the analysis of nanoindentation results [21,22,23,24,25,26]. 

The mechanical behaviors of the ZnO single crystal have been studied by some researchers using nanoindentation technology. Jian performed the nanoindentation test on a c-plane (0001) ZnO single crystal at the micro-scale indentation depth of about 1200 nm, and observed the multiple pop-ins phenomena in the load-displacement curves, which might have resulted from the sudden nucleation of dislocations [27]. Juday et al. studied the mechanical behaviors of an a-plane ($11\bar{2}0$) ZnO single crystal by nanoindentation at the submicron-scale indentation depth of about 500 nm [28]. Sung et al. examined the micro-scale mechanical characteristics on c-plane (0001), a-plane ($11\bar{2}0$) and m-plane ($10\bar{1}0$) ZnO single crystals by nanoindentation, a scanning electron microscope and cathodoluminescence spectroscopy [29]. Hirakata et al. investigated the initiation strength toplastic deformation of a c-plane (0001) ZnO single crystal by nanoindentation at the submicron-scale indentation depth of about 750 nm [30]. It can be seen that these studies carried out on a ZnO single crystal using nanoindentation technology mostly focus on the (0001) plane and micro/submicron scale, while limited studies on the mechanical behaviors at nano-scale were reported for the a-plane ($11\bar{2}0$) ZnO single crystal. 

During nanoindentation, the loading rate or strain rate is an important influencing factor on the mechanical properties of materials [31,32,33,34]. Kucheyev et al. studied the deformation behavior of a c-plane (0001) ZnO single crystal by nanoindentation at the micro-scale indentation depth of about 1000 nm, and found that the elastic–plastic-deformation transition threshold depends on the loading rate, with faster loading resulting in a larger threshold [35]. Lin et al. studied the creep behavior with different loading rates on c-plane (0001), a-plane ($11\bar{2}0$) and m-plane ($10\bar{1}0$) ZnO single crystals by nanoindentation experiments, and found that the creep displacements increased with the increasing loading rate [36]. Fang et al. studied the effect of strain rate on the hardness and Young’s modulus of ZnO thin films by nanoindentation [37]. Alsayed et al. investigated the mechanical properties of nano-ZnO at different strain rates using Vickers indentation tests [38]. However, there is little work on the understanding of how the strain rate affects the nanomechanical behavior of an a-plane ($11\bar{2}0$) ZnO single crystal.

In the present paper, nanoindentation tests were employed on the a-plane ($11\bar{2}0$) ZnO single crystal within the indentation depth of 100 nm, and the effect of strain rate on the mechanical behavior of the a-plane ($11\bar{2}0$) single crystal was investigated at the nano-scale level.

## 2. Experimental

### 2.1. Experimental Procedure

The bulk wurzite a-plane ($11\bar{2}0$) ZnO single crystal was provided by Shanghai Institute of Optics and Fine Mechanics, Chinese Academy of Sciences, and cut into a rectangular sample with dimensions of 10 × 5 × 0.5 mm^3^ susing the precise diamond-wire cutting machine. Prior to nanoindentation, the a-plane ($11\bar{2}0$) ZnO single crystal (10 × 5 mm^2^) was carefully polished to a near-mirror level. The nanoindentation tests were carried out using a nanoindentation System G200 (Agilent/Keysight Technologies, Inc., Santa Clara, CA, USA) with displacement and load resolutions of 0.01 nm and 50 nN, respectively. A triangular pyramid Berkovich diamond indenter with a tip radius of ~50 nm was employed. 

The nanoindentation tests in this study were carried out under three different strain rates of 0.01 s^−1^, 0.05 s^−1^ and 0.25 s^−1^. In nanoindentation, the strain rate, $\dot{\varepsilon }$, can be expressed as $\dot{\varepsilon }=\dot{h}/h$ [39], where *h* is the indentation depth and $\dot{h}$ is the instantaneous depth rate of the indenter. At each strain rate, the maximum indentation depths, *h*_max_, were set to be 10 nm, 20 nm, 40 nm, 60 nm, 80 nm and 100 nm. The indenter was loaded to the maximum load corresponding to the maximum depth during the loading stage, and held for 10 s during the holding stage to avoid the influence of creep on test results. After that, the load dropped to 10% of the maximum load for thermal-drift correction and then to zero. All tests were carried out at a room temperature of 25 °C, and the thermal drift was kept below 0.05 nm/s during the tests. The Poisson’s ratio of the a-plane ($11\bar{2}0$) ZnO single crystal was set to be 0.25 [29]. Eight indentations were made on the sample at each test condition to guarantee the repeatability of the test results. Each indent was separated by 5 μm to avoid any possible interference from the neighboring indents. Based on the Oliver–Pharr method and continuous-stiffness-measurement (CSM) technique, the hardness and Young’s modulus were measured continuously during the loading process.

### 2.2. Basic Principle

In the nanoindentation process, the indentation depth, *h*, and the corresponding indentation load, *P*, were obtained during one full cycle of the loading and unloading process. The Oliver–Pharr method [22] was applied to determine the hardness, *H*, as well as Young’s modulus, *E*. The hardness, *H*, of the specimen can be determined by:

(1) $$H=\frac{P}{A_{c}}$$

where *A*_c_ is the projection area of the contact zone between the indenter and workpiece. For a perfect Berkovich diamond indenter, the contact area, *A*_c_, is:

(2) $$A_{c}=24.56h_{c}^{2}$$

where *h*_c_ is the contact depth.

The Young’s modulus, *E*, of the specimen can be calculated from the following relationship:

(3) $$E=\left(1-\nu^{2}\right){\left[\frac{1}{E_{r}}-\frac{1-\nu_{\mathrm{tip}}^{2}}{E_{\mathrm{tip}}}\right]}^{-1}$$

where ν is the Poisson’s ratio of the specimen, and the *E*_tip_ and $\nu_{\mathrm{tip}}$ are the Young’s modulus and Poisson’s ratio of the Berkovich indenter (1141 GPa and 0.07), respectively; *E*_r_ is the reduced Young’s modulus, which is used to account for the combined response of the indenter and the specimen, and can be calculated by:

(4) $$E_{r}=\frac{S\sqrt{\pi }}{2\beta \sqrt{A_{c}}}$$

where *β* is the shape constant of the indenter (*β* = 1.034 for the Berkovich tip), *S* is the elastic contact stiffness, which is determined by the slope of the unloading curve, d*P*/d*h*. 

Based on the CSM technique, the *S* can be calculated as follows [40]:

(5) $$S={\left[\frac{1}{\left(P_{0}/z_{0}\right)cos\;\Phi -\left(K_{s}-m\omega^{2}\right)}-\frac{1}{K_{f}}\right]}^{-1}$$

where *P*_0_ is the amplitude of the harmonic-excitation force, $z_{0}$ is the magnitude of the resulting displacement oscillation, Φ is the phase angle between the force and displacement signals, ω is the frequency of the oscillation, $K_{s}$ is the spring constant of the leaf springs that support the indenter, *m* is the mass of the indenter, and $K_{f}$ is the stiffness of the indenter frame. 

Therefore, the contact stiffness, *S*, of the material can be obtained continuously at every interval of the load-depth curve in depth-controlled mode, which allows us to measure the hardness and Young’s modulus continuously over indentation depth.

## 3. Results and Discussion

### 3.1. Load-Indentation Depth Curves

**Figure 1:**
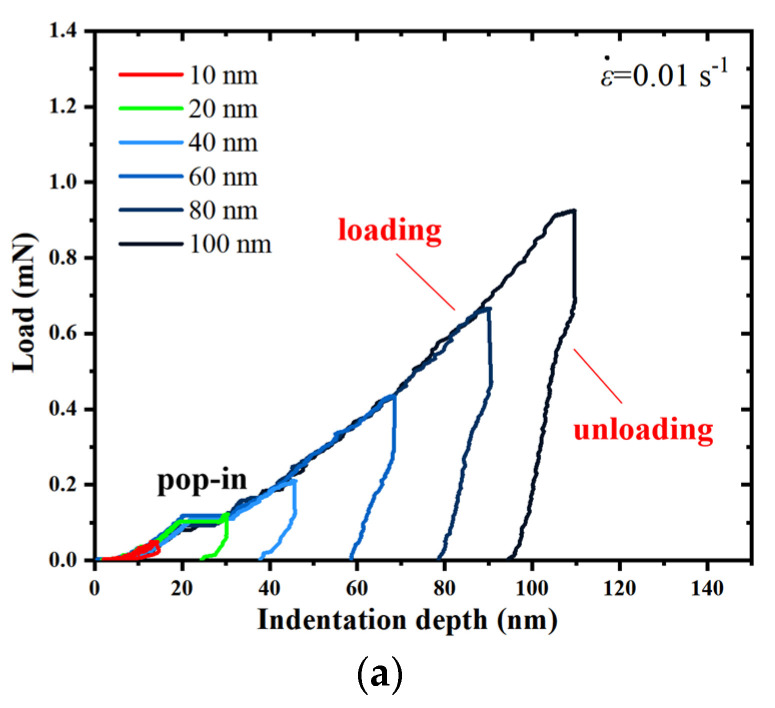

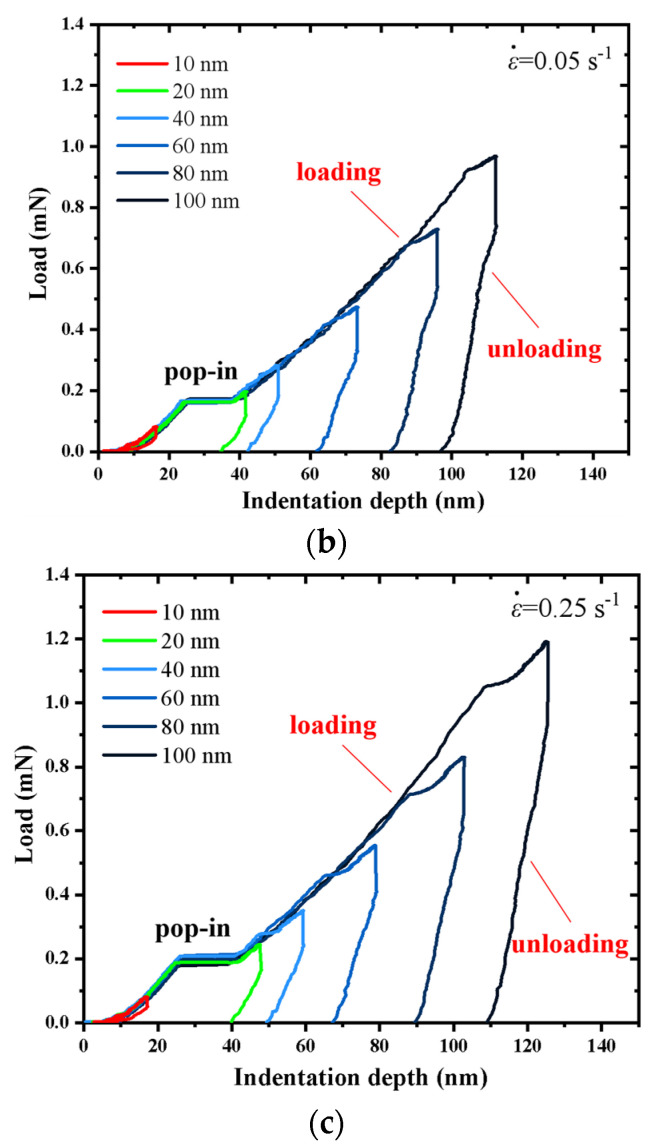
Typical load–indentation depth curves for a-plane ($11\bar{2}0$) ZnO single crystal at different indentation depths under the strain rates, $\dot{\varepsilon }$, of (**a**) 0.01 s^−1^, (**b**) 0.05 s^−1^, and (**c**) 0.25 s^−1^.

Figure 1a–c show the representative load-indentation depth curves of an a-plane ($11\bar{2}0$) ZnO single crystal at different maximum indentation depths, *h*_max_, under the strain rates, $\dot{\varepsilon }$, of 0.01 s^−1^, 0.05 s^−1^ and 0.25 s^−1^. It is observed that the loading curves under different indentation depths trace each other well, which reflects that the a-plane-($11\bar{2}0$)-ZnO-single-crystal sample is uniform and the nanoindentation test herein has a good repeatability. 

With the indentation depth increasing, the load increased gradually at each *h*_max_ during the loading process, and with the *h*_max_ increasing, the corresponding peak load also increased. It should be noted that a distinct depth burst, also known as pop-in event, occurred on each loading curve except that corresponding to the *h*_max_ of 10 nm. When the indentation depth reached the *h*_max_, the corresponding peak load was held for 10 s and then unloaded. During unloading, the depth decreased with the decreasing load. At the *h*_max_ of 10 nm corresponding to all the three strain rates, the indentation depths were almost fully recovered, indicating that only the elastic deformation occurred in the a-plane ($11\bar{2}0$) ZnO single crystal. However, at other indentation depths, there is a residual indentation depth after unloading, indicating that the irreversible plastic deformation occurred in the a-plane ($11\bar{2}0$) ZnO single crystal. It can be seen that the larger the peak load, the more the plastic deformation occurred in the indentation process of the a-plane ($11\bar{2}0$) ZnO single crystal at each strain rate.

**Figure 2:**
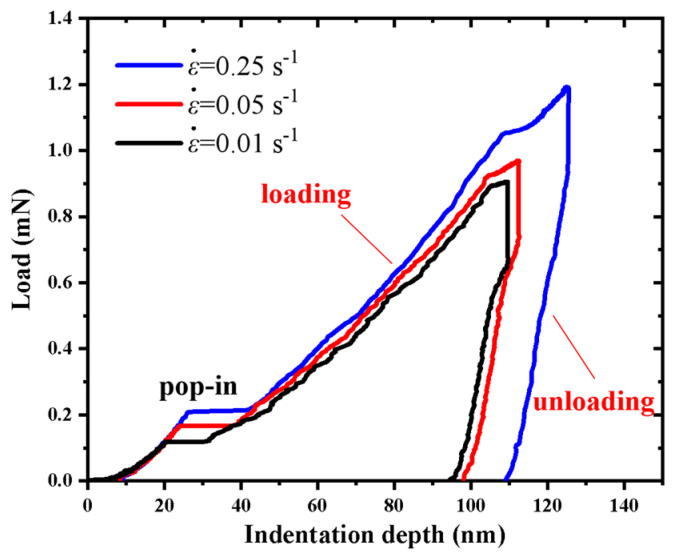
Comparison of the load–indentation depth curves at the indentation depth of 100 nm under three different strain rates.

In order to exhibit the effect of strain rate, the typical load-indentation depth curves at the *h*_max_ of 100 nm for the a-plane ($11\bar{2}0$) ZnO single crystal under three different strain rates are shown in Figure 2. It can be seen that the applied load at the same indentation depth increased with the increasing strain rate, showing the strain-rate sensitivity during the nanoindentation of the a-plane ($11\bar{2}0$) ZnO single crystal. Under higher strain rate, the indenter penetrates the material faster and the plastic deformation of the a-plane ($11\bar{2}0$) ZnO single crystal is not fully released, resulting in a greater applied load [41]. It is also noted that the actual maximum indentation depth increased with the increasing strain rate. Under the higher strain rate, more viscous deformation is accumulated after loading, transforming into larger creep deformation during the holding stage, leading to the greater actual indentation depth [42]. 

### 3.2. Pop-In

During the loading stage of nanoindentation, a sudden indentation-depth burst on a load–indentation-depth curve is referred to as a pop-in event, which has been observed in many different materials. An analysis of a pop-in event can provide valuable insights into the response of materials during deformation, such as the dislocation activity, elastic–plastic transition, and microcrack [43].

**Figure 3:**
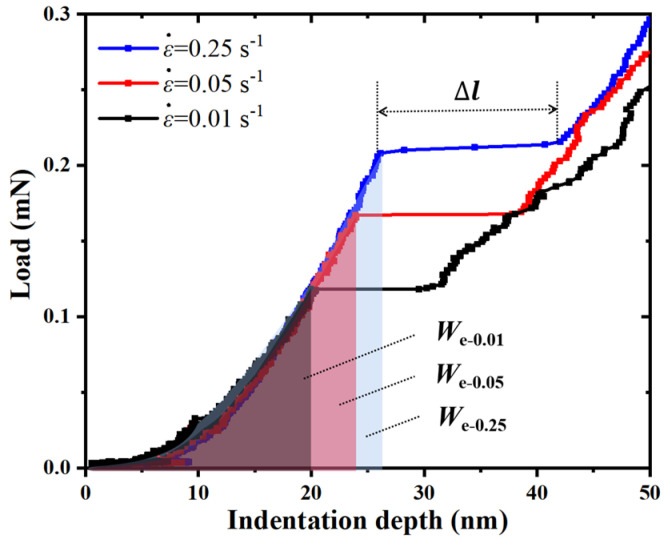
The pop-in events of the a-plane ($11\bar{2}0$) ZnO single crystal under three different strain rates.

**Figure 4:**
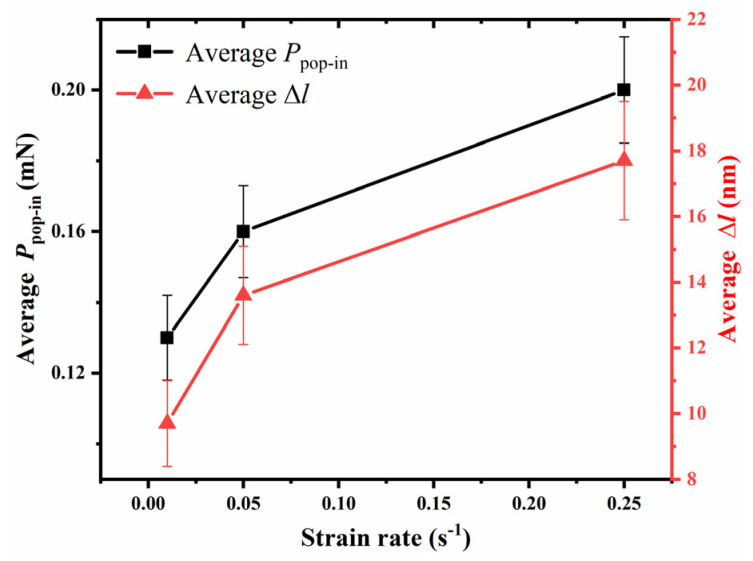
The average *P*_pop-in_ and average Δl corresponding to all the pop-in events at three strain rates in an a-plane ($11\bar{2}0$ ) ZnO single crystal.

To further observe the pop-in events at different strain rates in an a-plane ($11\bar{2}0$) ZnO single crystal, the 0–50 nm indentation-depth part of the load–indentation-depth curves corresponding to 100 nm maximum indentation depth at three strain rates are shown in Figure 3. It can be seen that there was distinct pop-in extension width, Δl, at each strain rate during the nanoindentation of the a-plane ($11\bar{2}0$) ZnO single crystal, and both the pop-in depth, *h*_pop-in_, and the corresponding pop-in load, *P*_pop-in_, increased with the increasing strain rate. The average *P*_pop-in_ and average Δl corresponding to all the pop-in events at three strain rates were also calculated, and their relationships with the strain rate are shown in Figure 4. It can be seen that when the strain rates were 0.01 s^−1^, 0.05 s^−1^, and 0.25 s^−1^, the average *P*_pop-in_ were 0.13 mN, 0.16 mN and 0.20 mN, respectively, and the average Δl were 9.7 nm, 13.6 nm and 17.7 nm, respectively. Both the *P*_pop-in_ and Δl increased with the increasing strain rate. Therefore, the pop-in load, pop-in depth and pop-in extension width all showed positive strain-rate sensitivities during the nanoindentation of an a-plane ($11\bar{2}0$) ZnO single crystal. 

Generally, the distinct pop-in event during nanoindentation marks the transition from elastic to plastic deformation, which is attributed to the dislocation nucleation in crystalline materials [44]. Before pop-in, a large amount of elastic energy can be stored, due to the lack of mobile defects, and upon the pop-in, the stored elastic energy is used for nucleating and moving a large number of dislocations, and is released by generating plastic deformation [45]. Therefore, the pop-in event is an indicator of the onset of plasticity. The yield stres, *τ*_y_, at the point of pop-in of a semiconductor crystal is generally described as a function of strain rate, $\dot{\varepsilon }$, and temperature, *T*, by the following empirical equation [46]:

(6) $$\tau_{y}=A{\dot{\varepsilon }}^{1/n}\exp\left(U/k_{B}T\right)$$

where *A* is a constant that depends on the crystal, *n* is a constant in the range of 2.59–4.44, *U* is a constant which represents a stress-independent activation energy, and is determined experimentally as 0.27 eV, and *k*_B_ is the Boltzmann constant [47]. 

According to Equation (6), the yield stress showed an increasing trend with the strain rat, so the corresponding pop-in load also increased with the increasing strain rate. The pop-in is a thermally activated event under loading. The lower strain rate leads to the increased time at load, which makes it easier for the critical thermal fluctuation to occur [48]. Therefore, the lower strain rate induced a lower pop-in load (corresponding to a smaller pop-in depth). 

As shown in Figure 3, the elastic-strain energy, *W*_e_, which is equal to the area under the elastic-loading curves also increased with the increasing strain rate (*W*_e-0.01_ *< W*_e-0.05_ *< W*_e-0.25_). According to the overall energy balance, the higher elastic-strain energy, *W*_e_, at the higher strain rate corresponds to the greater number of generated dislocation loops, *N.* The relation between the number of generated dislocation loops, *N*, and pop-in extension width, Δl, can be expressed as follows [49]:

(7) N=Δl/(2b)

where *b* is the magnitude of the Burgers vector, which was determined by the lattice parameter.

Based on this Equation (7), it can be found that Δl is proportional to *N*. Based on the proportional relationship between Δl and *N*, together with the positive correlation between the *N* and strain rate, it can be inferred that the pop-in extension width, Δl, at the higher strain rate was also larger during the nanoindentation of the a-plane ($11\bar{2}0$) ZnO single crystal. As a result, the lower strain rate made it easier to induce the pop-in event, while the higher strain rate deferred the pop-in event to higher load and deeper indentation depth, and made the pop-in extension width larger during the nanoindentation of the a-plane ($11\bar{2}0$) ZnO single crystal. 

### 3.3. Hardness and Young’s Modulus

**Figure 5:**
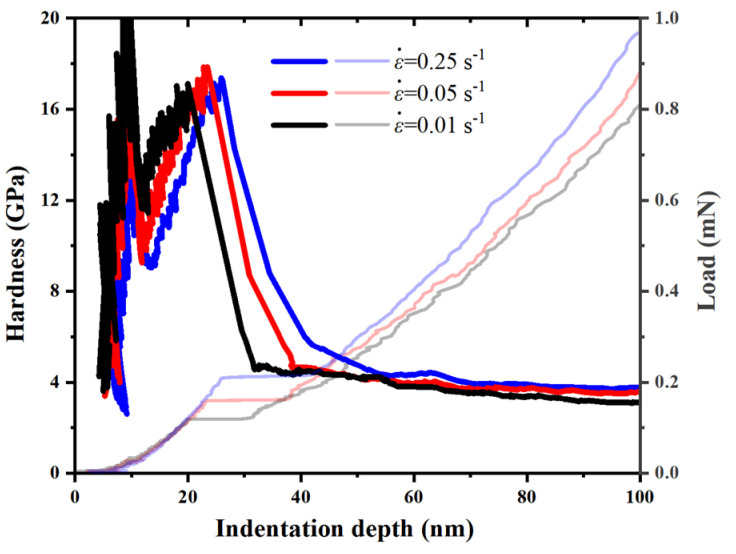
The hardness– indentation depth curves for the a-plane ($11\bar{2}0$) ZnO single crystal at the indentation depth of 100 nm under three different strain rates (The corresponding load–indentation depth curves are also displayed).

Figure 5 shows the typical hardness–indentation-depth curves and the corresponding load-indentation-depth curves of an a-plane ($11\bar{2}0$) ZnO single crystal at the maximum indentation depth of 100 nm, under the strain rates of 0.01 s^−1^, 0.05 s^−1^, and 0.25 s^−1^. It can be seen that the curves exhibited some fluctuations when the indentation depth was less than about 10 nm. This could be related to the resolution of the indenter, the smoothness of the specimen surface and the environmental noise for determining the initial contact position during the nanoindentation test [50]. When the indentation depth was more than 10 nm, the hardness at each strain rate increased rapidly and reached its peak at the onset of pop-in. Before the pop-in, the plastic deformation did not start, and the shear stress beneath the indenter increased with the increasing indentation depth, leading to the increased hardness [51]. During the pop-in, the hardness at each strain rate dropped quickly, which resulted from the dislocation nucleation and its avalanche-like multiplication [30,49]. The hardness decreased slowly after the pop-in, and then tended to be stable when the indentation depth was more than 80 nm. Generally, the phenomenon that the hardness decreases with the increasing indentation depth is known as normal indentation-size effect (ISE). Conversely, the phenomenon that the hardness increases with the increasing indentation depth is known as reverse ISE [52]. Therefore, the hardness of an a-plane ($11\bar{2}0$) ZnO single crystal showed reverse ISE before the pop-in, and exhibited normal ISE after the pop-in.

The average hardnesses of an a-plane ($11\bar{2}0$) ZnO single crystal under the three strain rates were calculated in the indentation-depth range of 80–100 nm, and their values corresponding to the strain rates of 0.01 s^−1^, 0.05 s^−1^ and 0.25 s^−1^ were 3.4 GPa, 3.7 GPa and 3.9 GPa, respectively. The hardness value of 3.9 GPa corresponding to the strain rate of 0.25 s^−1^ in our study is consistent with that reported by Sung et al. [29]. Compared with the c-plane (0001) ZnO single crystal, whose hardness was about 7.1 GPa [29,53], the a-plane ($11\bar{2}0$) ZnO single crystal is softer and has weaker resistance to the invasion of external objects. It can be seen that the hardness of the a-plane ($11\bar{2}0$) ZnO single crystal increased with the increasing strain rate, showing the strain-rate strengthening effect. Under the higher strain rate, there are fewer shear bands in the indentation area, so its inelastic deformation is smaller, and the hardness is higher [54,55].

**Figure 6:**
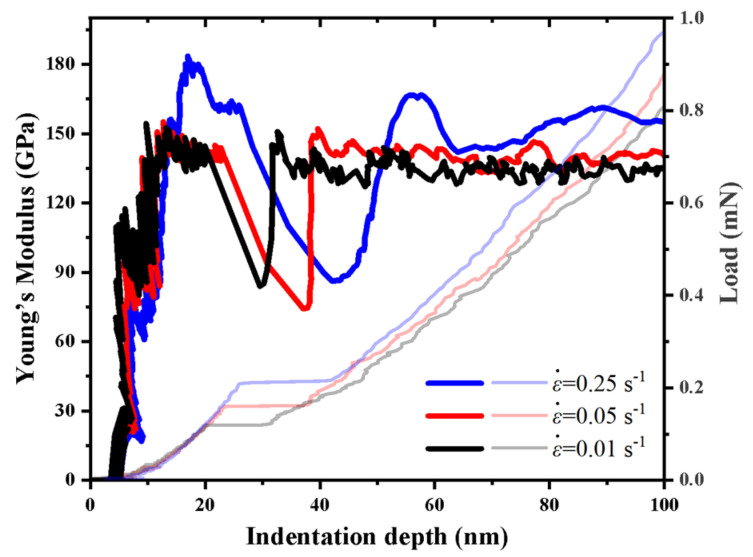
The Young’s modulus–indentation depth curves for the a-plane ($11\bar{2}0$) ZnO single crystal at the indentation depth of 100 nm, under three different strain rates (The corresponding load–indentation depth curves are also displayed).

Figure 6 shows the typical Young’s modulus–indentation-depth curves and the corresponding load–indentation-depth curves of the a-plane ($11\bar{2}0$) ZnO single crystal at the maximum indentation depth of 100 nm, under the strain rates of 0.01 s^−1^, 0.05 s^−1^ and 0.25 s^−1^. It can be seen that the Young’s modulus at each strain rate dropped suddenly, and then increased quickly during the pop-in. The sudden drop in Young’s modulus is due to the considerable scale of pop-in size caused by the great release of elastic energy during loading [56]. After the pop-in, the Young’s modulus of the a-plane ($11\bar{2}0$) ZnO single crystal at each strain rate showed no apparent dependency on the indentation depth.

The average Young’s moduli of the a-plane ($11\bar{2}0$) ZnO single crystal under the three strain rates were also calculated in the indentation-depth range of 80–100 nm. Their values corresponding to the strain rates of 0.01 s^−1^, 0.05 s^−1^ and 0.25 s^−1^ were 134 GPa, 142 GPa and 160 GPa, respectively. The Young’s modulus value of 160 GPa corresponding to the strain rate of 0.25 s^−1^ in our study is consistent with the results in the other literature [15,29]. The Young’s modulus of the a-plane ($11\bar{2}0$) ZnO single crystal is greater than that of the c-plane (0001) ZnO single crystal, which was reported as about 112 GPa [35,57]. Therefore, the a-plane ($11\bar{2}0$) ZnO single crystal is stiffer and has greater resistance to elastic deformation than the c-plane (0001) ZnO single crystal. It can be seen that the Young’s modulus of the a-plane ($11\bar{2}0$) ZnO single crystal increased with the increasing strain rate, exhibiting the positive strain-rate sensitivity. At the higher strain rate, there is no sufficient time for more lattice defects and dislocations to occur, which leads to the higher Young’s modulus [54,58].

## 4. Conclusions

In this paper, the nanoindentation tests of an a-plane ($11\bar{2}0$) ZnO single crystal were performed with six maximum indentation depths, *h*_max_, (10 nm, 20 nm, 40 nm, 60 nm, 80 nm and 100 nm) and three strain rates (0.01 s^−1^, 0.05 s^−1^ and 0.25 s^−1^). The load–indentation-depth curves, pop-in events, hardnesses and Young’s moduli were analyzed within 100 nm indentation depth, and their strain-rate sensitivity at a nano-scale level during the nanoindentation of an a-plane ($11\bar{2}0$) ZnO single crystal were investigated. The conclusions are summarized as follows:

1. With the indentation depth increasing, the load increased gradually at each maximum indentation depth, *h*_max_, during the loading process of the a-plane ($11\bar{2}0$) ZnO single crystal. A distinct pop-in event occurred on each loading curve, except that corresponding to the *h*_max_ of 10 nm. Only the elastic deformation occurred in the a-plane ($11\bar{2}0$) ZnO single crystal at the *h*_max_ of 10 nm, while at other *h*_max_, the irreversible plastic deformation emerged.
2. The applied load at the same indentation depth increased with the increasing strain rate, showing the positive strain-rate sensitivity during the nanoindentation of the a-plane ($11\bar{2}0$) ZnO single crystal. Due to the creep deformation increasing with strain rate during the holding stage, the actual maximum indentation depth at higher strain rate was greater.
3. The lower strain rate made it easier to induce the pop-in event of the a-plane ($11\bar{2}0$) ZnO single crystal, while the higher strain rate deferred the pop-in event to higher load and deeper indentation depth, and made the pop-in extension width larger. Therefore, the pop-in load, pop-in depth and pop-in extension width all showed positive strain-rate sensitivities during the nanoindentation of the a-plane ($11\bar{2}0$) ZnO single crystal.
4. The hardness of the a-plane ($11\bar{2}0$) ZnO single crystal showed reverse indentation size effect (ISE) before the pop-in, and exhibited normal ISE after the pop-in. Except during the pop-in, the Young’ modulus of the a-plane ($11\bar{2}0$) ZnO single crystal at each strain rate showed no apparent dependency on the indentation depth. Both the hardness and Young’s modulus of the a-plane ($11\bar{2}0$) ZnO single crystal increased with the increasing strain rate, exhibiting the positive strain-rate sensitivity.

## Data Availability

Not applicable.
